# A Complex Neurodevelopmental Phenotype Resembling a Chromatinopathy With Concurrent 7p Duplication and 10p Deletion Involving *ZMYND11* : A Case Report and Literature Review

**DOI:** 10.1002/mgg3.70164

**Published:** 2026-04-20

**Authors:** Elia Marco Paolo Minale, Stefania Martone, Chiara Criscuolo, Roberta Marra, Vito Alessandro Lasorsa, Raffaella Ruggiero, Teresa Suero, Mario Capasso, Immacolata Andolfo, Achille Iolascon, Roberta Russo, Michele Pinelli

**Affiliations:** ^1^ Department of Translational Medical Sciences University of Naples Federico II Naples Italy; ^2^ U.O.C. Medical Genetics, A.O.U. Federico II Naples Italy; ^3^ Department of Neurosciences, Reproductive Sciences and Odontostomatology University of Naples Federico II Naples Italy; ^4^ Center for Cognitive Disorders and Dementias—Neurology “Federico II” University Hospital Naples Italy; ^5^ Department of Molecular Medicine and Medical Biotechnology University of Naples Federico II Naples Italy; ^6^ CEINGE Biotecnologie Avanzate Franco Salvatore Naples Italy; ^7^ AMES, Centro Polidiagnostico Strumentale s.r.l. Naples Italy

**Keywords:** 10p15.3 microdeletion, 7p22.1 duplication, chromatinopathy, *ZMYND11*

## Abstract

**Background:**

The complex pathogenetic mechanisms of rare genetic diseases make the diagnostic process highly challenging. Advances in molecular genomic techniques, such as exome sequencing, have improved the identification of copy number variants (CNVs), increasing diagnostic yield.

**Methods:**

We report the case of a female patient with global developmental delay, growth alterations, and dysmorphic features. Clinical exome sequencing did not reveal point mutations. CNV analysis from exome data identified a 6 Mb microdeletion in 10p15.3p14, involving the *ZMYND11* gene, and a 7.6 Mb microduplication in 7p22.3p21.3; both rearrangements were subsequently confirmed by chromosomal microarray analysis. Conventional karyotyping revealed a derivative chromosome 10 [46,XX,der (10)], a finding consistent with the possibility of an unbalanced translocation involving chromosomes 7 and 10. The combined cytogenetic and molecular findings are consistent with the possibility that the duplicated 7p segment is inserted into the short arm of chromosome 10.

**Results:**

*ZMYND11*, a dosage‐sensitive gene, has been associated with Cornelia de Lange Syndrome (CdLS)‐like phenotypes, and its haploinsufficiency is linked to 10p15.3 microdeletion syndrome. Our patient presented a complex phenotype due to the concurrent 7p duplication and 10p deletion, highlighting the importance of *ZMYND11* in chromatinopathies. A review of similar cases supports considering *ZMYND11* in evaluating chromatinopathy‐related features. Notably, she also exhibited unique characteristics that have not been previously described in association with either CNV.

**Conclusion:**

Next‐generation sequencing, capable of detecting both single nucleotide variants and CNVs, is a critical tool for diagnosing neurodevelopmental disorders and uncovering diverse causative variants. This case emphasizes how NGS facilitates the identification of co‐occurring CNVs and expands the phenotypic spectrum associated with chromatinopathies. Detailed characterization of such complex phenotypes using NGS is essential for advancing our understanding of rare genetic conditions and improving diagnostic accuracy.

## Graphical abstract

This study presents a 25‐year‐old female with global developmental delay, dysmorphic features, and a complex phenotype arising from co‐occurring chromosomal anomalies: a 6 Mb 10p15.3p14 microdeletion involving the ZMYND11 gene and a 7.6 Mb 7p22.3p21.3 microduplication. The findings emphasize the role of ZMYND11 in chromatinopathies and highlight the diagnostic power of next‐generation sequencing in uncovering concurrent CNVs, broadening the phenotypic spectrum of these genetic conditions. This case underscores the necessity of comprehensive genomic analyses to refine our understanding of rare genetic disorders.

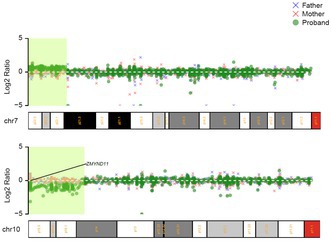

## 1 Introduction

The molecular diagnosis of diseases with genetic heterogeneity is challenging, particularly for neurodevelopmental disorders (NDDs), including chromatinopathies (CPs). CPs are conditions characterized by complete penetrance and de novo onset, and their pathogenic mechanism involves haploinsufficiency. Genomic mutations impact dose‐sensitive genes, including *loss‐of‐function* mutations (e.g., frameshift, nonsense, missense, or splicing mutations) or chromosomal deletions, causing chromatin epigenetic deregulation. Clinically, patients exhibit intellectual disability, growth alterations, and distinct dysmorphic features. Pathogenic variants in *NIPBL* (OMIM #122470), *SMC1A* (OMIM #300590), *SMC3* (OMIM #610759), *HDAC8* (OMIM #300882), and *RAD21* (OMIM #614701) cause Cornelia de Lange Syndrome (CdLS). Many genes have been associated with CPs classified as CdLS in patients with a milder phenotype (Avagliano et al. 2020). CdLS is now seen as a spectrum, from classic CdLS presentation to similar, non‐classic appearance, caused by pathogenic variants in genes involved in cohesin functioning (Kline et al. 2018). Among a cohort of 57 patients clinically diagnosed with CdLS, one carried a truncating mutation in *ZMYND11* (OMIM #616083) (Aoi et al. 2019). *ZMYND11* encodes a transcription corepressor (Wen et al. 2014), and missense mutations in this gene are associated with severe neurodevelopmental disorder phenotype (Cobben et al. 2014). Haploinsufficiency of *ZMYND11* drives the 10p15.3 microdeletion syndrome, linked to NDD, behavioral disturbances, hypotonia, seizures, low birth weight, short stature, genitourinary malformations, and characteristic facial features (Tumiene et al. 2017).

We report a female patient with a neurodevelopmental disorder, growth alterations, and dysmorphic features resembling CP. A 6 Mb microdeletion at 10p15.3p14, including *ZMYND11*, likely explains her phenotype and supports the chromatinopathy hypothesis. This case also expands the known phenotypic variability associated with *ZMYND11* haploinsufficiency, suggesting broader clinical manifestations beyond the classical 10p15.3 microdeletion syndrome. The patient also exhibited a 7.6 Mb microduplication at 7p22.3p21.3, contributing to the specific features observed in her. Karyotype further revealed a derivative chromosome 10, supporting the hypothesis of a complex unbalanced rearrangement underlying the concurrent CNVs. This case illustrates a complex phenotype arising from the combined effects of two rare CNVs and suggests that structural rearrangements may contribute to the resulting clinical variability.

## 2 Methods

### 2.1 Ethical Compliance

Clinical and genetic investigations were performed in accordance with the Declaration of Helsinki. Written informed consent for publication was obtained from the patient and her parents.

**FIGURE 1:**
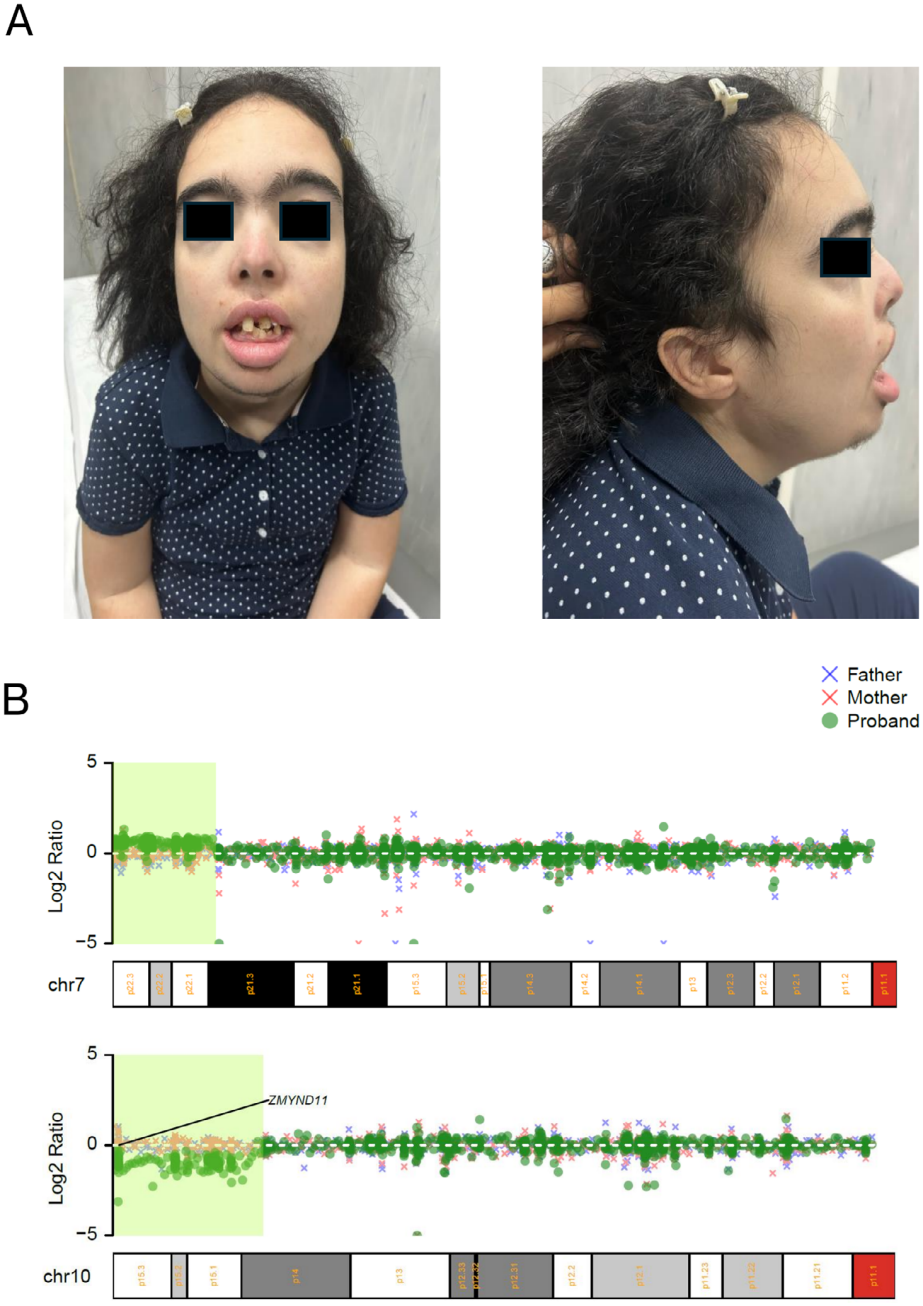
(A) The figure presents a frontal and a lateral view of the patient. The frontal view highlights arched and thick eyebrows, synophrys, a broad nasal bridge, thick lips, the presence of supernumerary teeth, and hypertrichosis of the face. The lateral view primarily shows low‐set, posteriorly rotated ears. (B) The figure shows the log_2_ ratio of the test sample's read depths normalized to the pooled reference. Detailed view of chromosome 7, carrying a duplication involving the band p22, and chromosome 10, carrying a deletion involving the band p15, with the *ZMYND11* gene highlighted. The log_2_ ratios of the three family members are overlaid.

Our patient, a 25‐year‐old Italian female, only childof non‐consanguineous parents, presented at birth with a cleft palate, bifid uvula, congenital torticollis, bilateral vesicoureteral reflux, right kidney hydronephrosis, and patent ductus arteriosus, which closed spontaneously by age two. She spoke at three, walked at six, gained sphincter control by seven. She attended school up to 3rd grade with support, recognizing numbers and letters but unable to read or write. Sociable and responsive, she communicates through gestures and sounds. Menarche occurred at 13 with regular cycles. She had postnatal growth retardation, episodes of absence seizures, and chewing difficulties. No family history of developmental delay up to second‐degree relatives. At 25, she was admitted to our Medical Genetics Unit. She presented with short stature (130 cm, < 1st percentile, −5.1 SD), low weight (38 kg, < 1st percentile, −2.9 SD), and a head circumference of 53.5 cm (22nd percentile). Features included synophrys, thick arched eyebrows, long eyelashes, downslanting palpebral fissures, low‐set ears, broad nasal bridge, thick lips, retrognathia, supernumerary teeth, and a normal palate with a postsurgical scar (Figure 1A). She had hypertrichosis on her legs, trunk, and face, scoliosis, wrist movement limitations, nighttime drooling, was nonverbal, and had walking difficulties. The patient's short stature, intellectual delay, hirsutism, and facial features suggested a CdLS spectrum disorder. Her clinical CdLS score of 4 met the criteria for molecular testing (Kline et al. 2018). A trio‐clinical exome was conducted. Genomic DNA extraction and exome analysis utilized a commercial panel covering over 5000 genes linked to hereditary diseases (SureSelect custom Constitutional Panel 17 Mb, Agilent Technologies) (Martone et al. 2022). Variant pathogenicity was assessed using ACMG/AMP guidelines (Tavtigian et al. 2020). Initial SNV analysis found no pathogenic/likely pathogenic variants or variants of unknown significance in CdLS‐related or other syndrome‐causing genes. The second‐tier analysis, using CNVkit (v0.9.9) (Talevich et al. 2016), identified a de novo 10p15.3 microdeletion and a de novo 7p22.3 microduplication in the patient (Figure 1B). CNV detection was achieved by comparing test sample read depths to a pooled reference of over 200 samples from our Institute (Pfundt et al. 2017), followed by combining CNV profiles of all three family members. CNVs were annotated using AnnotSV (v3.0.7), and diagrams were created with the R package “Karioploter” (Gel and Serra 2017). Further analysis with a single nucleotide polymorphism array (HumanCytoSNP‐12 BeadChip, Illumina) defined the boundaries of the genomic rearrangements: a 7.6 Mb microduplication in 7p22.3p21.3 (chr7:160,803‐7,788,332;hg19;GRCh37) and a 6 Mb microdeletion in 10p15.3p14 (chr10:93,705‐7,276,215;hg19;GRCh37). Karyotype (GTG banding, 550‐band resolution) was performed on the proband and her father. The patient's exam revealed a derivative chromosome 10, described as 46,XX,der (10), indicating the presence of additional material on the short arm of chromosome 10. This finding was consistent with the duplication of chromosome 7p22.3p21.3 identified by SNP‐array. The father's karyotype was normal (46,XY), and the mother was not available for cytogenetic evaluation. FISH analysis was recommended to confirm the suspected insertion of the duplicated 7p segment into the derivative chromosome 10. However, the test could not be performed due to the patient's unavailability for additional blood sampling.

## 3 Results

The 10p15.3p14 deletion and 7p22.3p21.3 duplication affected the critical regions for 10p15.3 microdeletion and 7p22.1 microduplication syndromes, respectively. Karyotype revealed a derivative chromosome 10 [46,XX,der (10)], suggesting the presence of duplicated 7p material on the short arm of chromosome 10. This finding provides evidence for a probable unbalanced translocation between chromosomes 7 and 10. Despite the lack of FISH in structural analysis, the rearrangement configuration can be reliably established based on the combined molecular and cytogenetic data. The 10p15.3 microdeletion syndrome is characterized by neurodevelopmental disorders, seizures, genitourinary malformations, hypotonia, short stature, low birth weight, and facial dysmorphisms (Eggert et al. 2016; Poluha et al. 2017; Chen et al. 2019). *ZMYND11* haploinsufficiency is considered the primary pathogenic mechanism, as clinical differences are minimal between large and small 10p15.3 deletions when *ZMYND11* is involved (Eggert et al. 2016; Poluha et al. 2017; Chen et al. 2019; Shao et al. 2021), including *ZMYND11* intragenic deletions (Chen et al. 2019) and truncating mutations (Coe et al. 2014; Popp et al. 2017; Aoi et al. 2019). The deleted region in our patient included at least 8 known RefSeq genes, including *ZMYND11* (S1). The 7p22.1 microduplication syndrome is associated with neurodevelopmental disorders, cardiac and skeletal abnormalities, and facial dysmorphisms, with duplication sizes reported between 886 Kb and 2.4 Mb (Caselli et al. 2015; Goitia et al. 2015). In our patient, the 7.6 Mb duplication at 7p22.3p21.3 encompassed the critical region for 7p22.1 duplication syndrome, including at least 20 known RefSeq genes (S2). We systematically reviewed cases of 10p15.3 microdeletions, *ZMYND11* truncating mutations, and 7p22.1 microduplications, totaling 61 patients (Table 1). Our patient's clinical profile was then compared with these cases. Notably, our patient showed no head or eye abnormalities. In the literature, macrocephaly and hypertelorism are common in 7p22.1 duplication cases, while some patients with 10p15.3 deletions or *ZMYND11* mutations have microcephaly. Our patient presented dysmorphic features seen in deletion cases (e.g., arched eyebrows, synophrys), duplication cases (e.g., downslanting palpebral fissures, forehead anomalies), or both (e.g., low‐set ears). She exhibited supernumerary teeth, consistent with dental anomalies in some 10p15.3 microdeletion or *ZMYND11* mutation cases. Additionally, she had a bifid uvula, a previously unreported feature, and cleft palate, noted in only one patient with 7p22.1 duplication. Global developmental delay was present in all reviewed cases, including our patient. Autistic behavior, which was also present in our patient, was observed in 61% of *ZMYND11* mutation cases, 10% of 10p15.3 microdeletions, and one 7p22.1 duplication case (16%). Our patient experienced absence seizures, consistent with 69% of *ZMYND11* mutation cases and 65% of 10p15.3 microdeletion cases, though seizures have not been linked to 7p22.1 duplication. She also had a patent ductus arteriosus (PDA), observed in one‐third of 7p22.1 duplication cases, which also featured minor heart anomalies. Tetralogy of Fallot was reported in one 10p15.3 deletion case, with no cardiac issues linked to *ZMYND11* mutations. Short stature, seen in some *ZMYND11* mutation and 10p15.3 deletion cases, was also present in our patient, while no growth issues were reported in 7p22.1 duplication. Her urinary anomaly aligns with sporadic cases across all groups in the literature, and she exhibited hypertrichosis, a feature common in some chromatinopathies but previously unreported in these conditions. The presence of a structurally rearranged chromosome 10 may help explain the atypical combination of clinical features, as well as the co‐occurrence of both CNVs, supporting the interpretation of a complex unbalanced rearrangement involving the insertion of 7p material into 10p.

**TABLE 1:** Comparative analysis of clinical and genetic findings of 10p15.3 deletions, *ZMYND11* truncating variants, and 7p22.1 duplications.

Literature (References)		10p15.3 deletions (*N* = 29 patients)	*ZMYND11* variants (*N* = 26 patients)	7p22.1 duplications (*N* = 6 patients)	TOT (*N* = 61 patients)	Our patient
		11,12,13,14	3,5,15,16	17,18		
Head abnormalities	Macrocephaly	0/29 (0%)	1/26 (3%)	5/6 (83%)	6/61 (9%)	−
	Microcephaly	2/29 (6%)	1/26 (3%)	0/6 (0%)	3/61 (4%)	−
Eye abnormalities	Hypertelorism	2/29 (6%)	0/26 (0%)	5/6 (83%)	7/61 (11%)	−
Facial abnormalities	Abnormality of the forehead	0/29 (0%)	0/26 (0%)	3/6 (50%)	3/61 (5%)	+
	Highly arched eyebrow	21/29 (72%)	1/26 (3%)	0/6 (0%)	22/61 (36%)	+
	Synophrys	1/29 (3%)	2/26 (7%)	0/6 (0%)	3/61 (5%)	+
	Downslanting palpebral fissures	0/29 (0%)	1/26 (3%)	3/6 (50%)	4/61 (6%)	+
	Low‐set ears	24/29 (82%)	2/26 (7%)	5/6 (83%)	31/61 (50%)	+
	Broad nasal bridge	5/29 (17%)	2/26 (7%)	1/6 (16%)	8/61 (13%)	+
	Abnormal mandible morphology	3/29 (10%)	2/26 (7%)	2/6 (33%)	7/61 (11%)	+
Abnormal oral cavity morphology	Abnormality of the dentition	4/29 (13%)	1/6 (3%)	0/6 (0%)	5/61 (8%)	+
	Cleft palate	0/29 (0%)	0/26 (0%)	1/6 (16%)	1/61 (1%)	+
	Bifid uvula	0/29 (0%)	0/26 (0%)	0/6 (0%)	0/61 (0%)	+
Abnormality of the nervous system	Global developmental delay	29/29 (100%)	26/26 (100%)	6/6 (100%)	61/61 (100%)	+
	Autistic behavior	3/29 (10%)	16/26 (61%)	1/6 (16%)	20/61 (32%)	+
	Seizures	19/29 (65%)	18/26 (69%)	0/6 (0%)	4/61 (60%)	+
Congenital heart defect	Atrial septal defect	0/29 (0%)	0/26 (0%)	3/6 (50%)	3/61 (5%)	−
	Patent ductus arteriosus (PDA)	0/29 (0%)	0/26 (0%)	2/6 (33%)	2/61 (3%)	+
	Tetralogy of Fallot	1/29 (3%)	0/26 (0%)	0/6 (0%)	1/61 (1%)	−
	Patent foramen ovale	0/29 (0%)	0/26 (0%)	2/6 (33%)	2/61 (3%)	−
Abnormality of the genitourinary system	Cryptorchidism	1/29 (3%)	1/26 (3%)	5/6 (83%)	7/61 (11%)	NA
	Abnormality of the urinary system	1/29 (3%)	1/26 (3%)	1/6 (16%)	3/61 (5%)	+
Other	Short stature	1/29 (3%)	7/26 (26%)	0/6 (0%)	8/61 (13%)	+
	Hypertrichosis	0/29 (0%)	0/26 (0%)	0/6 (0%)	0/61 (0%)	+

*Note:* The table summarizes clinical features observed in our patient and in individuals with 10p15.3 deletions, *ZMYND11* mutations, and 7p22.3 duplications. It highlights the prevalence of abnormalities across various body systems. Data are presented as counts and percentages, showing the manifestation of traits according to different genetic alterations and indicating the presence in affected individuals. NA, not available; + reported; − not reported.

## 4 Conclusion

In summary, our patient exhibited a complex mix of clinical features from several known syndromes, along with some unique traits. These findings suggest that the *ZMYND11* gene should be included in diagnostic evaluations for patients with chromatinopathy‐related features (Aoi et al. 2019; Shangguan and Chen 2022). The co‐occurrence of a deletion and a duplication in our patient, likely resulting from a complex structural rearrangement, highlights the importance of comprehensive genetic analysis, especially when the phenotype does not fit classical syndromic categories. In our case, the presence of a derivative chromosome 10 provided structural evidence supporting a unified origin for the co‐occurring CNVs. While haploinsufficiency of *ZMYND11* has been linked to 10p15.3 microdeletion syndrome, emerging evidence, including our case, points to a broader phenotypic spectrum associated with disruptions of this gene. Longitudinal studies of patients with similar chromosomal anomalies are needed to clarify phenotypic variability and improve diagnostic and management guidelines. NGS, which captures both CNVs and SNVs in one assay, is increasingly favored for diagnosing neurodevelopmental disorders due to its higher diagnostic yield and comprehensive insight into genetic causes.

## Author Contributions

E.M.P.M. and S.M. contributed equally to this work. E.M.P.M. and S.M. contributed to study conception/design, data acquisition and interpretation, and manuscript drafting. M.P. supervised the study and critically revised the manuscript. All authors contributed to data collection/interpretation, critically revised the manuscript for important intellectual content, and approved the final version.

## Funding

This work was supported by the Italian Ministry of Health (Ministero della salute, Italy) under the National Recovery and Resilience Plan (PNRR), financed by the NextGenerationEU (grant PNRR‐MR1‐2022‐12376067; CUP: C63C22001390007; “Rafforzamento e potenziamento della ricerca biomedica del SSN”).

## Conflicts of Interest

The authors declare no conflicts of interest.

## Supporting information


**Data S1:** Eight RefSeq genes encompassed in 10p15.3 deletion.

**Data S2:** Twenty RefSeq genes encompassed in 7p22.1 duplication.

## Data Availability

All data generated or analyzed during this study are included in this published article and its Supporting Information files.
