# Supplementary material for: A Complex Neurodevelopmental Phenotype Resembling a Chromatinopathy With Concurrent 7p Duplication and 10p Deletion Involving ZMYND11 : A Case Report and Literature Review
Source: Mol Genet Genomic Med. 2026 Apr 20;14(4):e70164. doi: 10.1002/mgg3.70164 (PMC13093532; doi:10.1002/mgg3.70164)
Supplement: Supplementary file 1 — Data S1: Eight RefSeq genes encompassed in 10p15.3 deletion. [file MGG3-14-e70164-s002.docx]

**S1. Eight RefSeq genes encompassed in 10p15.3 deletion**

| Genes (NM) | Inheritance | ClinGen Haploinsufficiency | Calculated LOF score^1^ | pLI^2^ | LOF (upper)^3^ | Decipher HI score^4^ | OMIM condition associated |
| --- | --- | --- | --- | --- | --- | --- | --- |
| TUBB8  (NM_177987) | AD, AR, Semi Dominant | N/A | 2 | 0 | 1.29 | 89.22 | Oocyte maturation defect 2 |
| PITRM1  (NM_014889) | AR | N/A | 1 | 0 | 1 | 75.61 | Spinocerebellar Ataxia, Autosomal Recessive 30 |
| IL2RA  (NM_000417) | AR | AR | 3 | 0 | 0.91 | 67.75 | Immunodeficiency Due To CD25 Deficiency; Type 1 Diabetes Mellitus 10 |
| AKR1C2  (NM_205845) | AR | N/A | 0 | 0 | 1.04 | 90.47 | 46,XY Disorder Of Sex Development Due To Testicular 17,20-Desmolase Deficiency |
| AKR1C4  (NM_001818) | AR | N/A | 0 | 0 | 1.34 | 88.97 | 46,XY Disorder Of Sex Development Due To Testicular 17,20-Desmolase Deficiency |
| ZMYND11  (NM_001370100) | AD | 3 | 3 | 1 | 0.17 | 16.99 | Intellectual Disability, Autosomal Dominant 30 |
| WDR37  (NM_014023) | AD | N/A | 0 | 1 | 0.42 | 26.42 | Neurooculocardiogenitourinary Syndrome |
| KLF6  (NM_001300) | N/A | N/A | 1 | 1 | 0.11 | 17.75 | Familial Prostate Carcinoma; Gastric Cancer |
| ASB13  (NM_024701) | N/A | N/A | 0 | 0 | 1.69 | 57.74 | - |
| PITRM1-AS1  (NR_038284) | N/A | N/A | 0 | N/A | N/A | N/A | - |
| LINC00700  (NR_040253) | N/A | N/A | 0 | N/A | N/A | N/A | - |
| ADARB2-AS1  (NR_033387) | N/A | N/A | 0 | N/A | N/A | N/A | - |
| LARP4B  (NM_015155) | N/A | N/A | 0 | 1 | 0.29 | 57.09 | - |
| GTPBP4  (NM_012341) | N/A | N/A | 0 | 1 | 0.41 | 36.18 | - |
| IDI1  (NM_004508) | N/A | N/A | 0 | 0.04 | 0.91 | 69.3 | - |
| LINC00705  (NR_015425) | N/A | N/A | 0 | N/A | N/A | N/A | - |
| AKRIE2  (NM_001040177) | N/A | N/A | 0 | 0 | 1.3 | 87.28 | - |
| LINC02657  (NR_134491) | N/A | N/A | 0 | N/A | N/A | N/A | - |
| CALML5  (NM_017422) | N/A | N/A | 0 | 0.01 | 1.88 | 96.2 | - |
| CALML3  (NM_005185) | N/A | N/A | 0 | 0.01 | 1.71 | 63.41 | - |
| ADARB2  (NM_018702) | N/A | N/A | 0 | 0 | 0.68 | 43.56 | - |
| LINC02645  (NR_136146) | N/A | N/A | 0 | N/A | N/A | N/A | - |
| LINC00200 (NR_015376) | N/A | N/A | 0 | N/A | N/A | N/A | - |
| SNORD142  (NR_132758) | N/A | N/A | 0 | N/A | N/A | N/A | - |
| NET1 (NM_001047160) | N/A | N/A | 0 | 0 | 1.03 | 50.99 | - |
| UCN3 (NM_053049) | N/A | N/A | 0 | 0 | 1.74 | 75.53 | - |
| AKR1C3  (NM_003739) | N/A | N/A | 0 | 0 | 1.34 | 89.81 | - |
| TASOR2 (NM_001321783) | N/A | N/A | 0 | N/A | N/A | 71.24 | - |
| MIR6078  (NR_106726) | N/A | N/A | 0 | N/A | N/A | N/A | - |
| PFKP-DT (NR_160682) | N/A | N/A | 0 | N/A | N/A | N/A | - |
| ANKRD16  (NM_019046) | N/A | N/A | 0 | N/A | 1.36 | 73.25 | - |
| LOC101927964  (NR_120632) | N/A | N/A | 0 | N/A | N/A | N/A | - |
| LINC02561  (NR_149060) | N/A | N/A | 0 | N/A | N/A | N/A | - |
| DIP2C  (NM_014974) | N/A | N/A | 0 | 1 | 0.24 | 41.18 | - |
| LOC101930421  (NR_147612) | N/A | N/A | 0 | N/A | N/A | N/A | - |
| AKR1C1 (NM_001353) | N/A | N/A | 0 | 0 | 1.26 | 91.68 | - |
| LINC00701  (NR_038884) | N/A | N/A | 0 | N/A | N/A | N/A | - |
| AKR1C6P  (NR_026743) | N/A | N/A | 0 | N/A | N/A | N/A | - |
| IL15RA  (NM_002189) | N/A | N/A | 0 | 0 | 1.46 | 86.89 | - |
| IDI2  (NM_033261) | N/A | N/A | 0 | 0 | 1.58 | 99.25 | - |
| LINC00703  (NR_108054) | N/A | N/A | 0 | N/A | N/A | N/A | - |
| LINC00702  (NR_108040) | N/A | N/A | 0 | N/A | N/A | N/A | - |
| IDI2-AS1  (NR_024629) | N/A | N/A | 0 | N/A | N/A | N/A | - |
| LOC105376360  (NR_131187) | N/A | N/A | 0 | N/A | N/A | N/A | - |
| GDI2  (NM_001494) | N/A | N/A | 0 | 0.47 | 0.78 | 20.83 | - |
| AKR1C8P  (NR_027916) | N/A | N/A | 0 | N/A | N/A | N/A | - |
| LOC101927762  (NR_120629) | N/A | N/A | 0 | N/A | N/A | N/A | - |
| PFKP  (NM_002627) | N/A | N/A | 0 | 0 | 1.29 | 68.91 | - |
| LINC02639  (NR_134490) | N/A | N/A | 0 | N/A | N/A | N/A | - |
| MIR5699  (NR_049884) | N/A | N/A | 0 | N/A | N/A | N/A | - |
| TUBAL3  (NM_024803) | N/A | N/A | 0 | 0 | 1.46 | 80.16 | - |
| MIR6072  (NR_106720) | N/A | N/A | 0 | N/A | N/A | N/A | - |
| LINC02669  (NR_155744) | N/A | N/A | 0 | N/A | N/A | N/A | - |
| LOC105376353  (NR_164119) | N/A | N/A | 0 | N/A | N/A | N/A | - |
| FBH1  (NM_178150) | N/A | N/A | 0 | 1 | 0.45 | 57.79 | - |
| MANCR  (NR_024475) | N/A | N/A | 0 | N/A | N/A | N/A | - |
| CALML3-AS1  (NR_12049) | N/A | N/A | 0 | N/A | N/A | N/A | - |

1.Calculated LOF (loss of function) score: Franklin calculated loss of function score, based on the pathogenic/likely pathogenic null variants reported in this gene. 5 or more null P/LP reported variants in the gene are considered strong evidence that LOF is a mechanism of disease for this gene, 2-4 are considered moderate evidence, while a single variant is considered as limited evidence.

2.pLI: Probability of Loss-of-function Intolerance. Genes with higher numbers are more likely to be dosage sensitive. The probability that a gene is intolerant to loss-of-function mutations based on the gene's observed depletion of LOF variants in gnomAD. Genes with high pLI scores (pLI ≥ 0.9) are extremely LoF intolerant, whereby genes with low pLI scores (pLI ≤ 0.1) are LoF tolerant

3.LOF Loss-of-function Observed / Expected Upper bound Fraction. Genes with lower numbers are more likely to be dosage sensitive. A quantitative measure of the observed depletion (or enrichment) of loss-of-function variants in gnomAD compared to a null mutational model. The minimum value is 0, but there is theoretically no maximum value. Genes with smaller values (closer to zero) are more intolerant of mutations. Genes with a score of less then 0.35 are presented in red and are more likely to be haploinsufficient.

4.Decipher HI (HaploInsufficiency) Score: Probability of being a haploinsufficient gene. Genes with a lower percentage are more likely to be dosage sensitive. Percentages refer to genome-wide percentiles of genes ranked according to their haploinsufficient score. High ranks (e.g. 0-10%) indicate a gene is more likely to exhibit haploinsufficiency, low ranks (e.g. 90-100%) indicate a gene is more likely to NOT exhibit haploinsufficiency.

5. N/A not available; AR autosomal recessive; AD autosomal dominant
