# Supplementary material for: A Complex Neurodevelopmental Phenotype Resembling a Chromatinopathy With Concurrent 7p Duplication and 10p Deletion Involving ZMYND11 : A Case Report and Literature Review
Source: Mol Genet Genomic Med. 2026 Apr 20;14(4):e70164. doi: 10.1002/mgg3.70164 (PMC13093532; doi:10.1002/mgg3.70164)
Supplement: Supplementary file 2 — Data S2: Twenty RefSeq genes encompassed in 7p22.1 duplication. [file MGG3-14-e70164-s001.docx]

**S2. Twenty RefSeq genes encompassed in 7p22.1 duplication.**

| Genes (NM) | Inheritance | ClinGen Triplosensitivity | OMIM condition associated |
| --- | --- | --- | --- |
| ACTB  (NM_001101) | AD | 0 | BaraitserWinter Syndrome 1, ACTBassociated Syndromic Thrombocytopenia |
| BRAT1  (NM_152743) | AR | 0 | Neonatalonset Encephalopathy With Rigidity And Seizures |
| IQCE  (NM_152558) | AR | AR | Polydactyly Postaxial Type A7 |
| RNF216  (NM_207111) | AR | N/A | cerebellar ataxiahypogonadism syndrome |
| CARD11  (NM_032415) | AD, AR | N/A | BENTA disease, immunodeficiency 11b with atopic dermatitis, severe combined immunodeficiency due to CARD11 deficiency |
| GET4  (NM_015949) | AR | N/A | congenital disorder of glycosylation type IIy |
| MRM2  (NM_013393) | AR | N/A | mitochondrial DNA depletion syndrome 17 |
| RAC1  (NM_001300) | AD | N/A | intellectual disability autosomal dominant 48 |
| KDELR2  (NM_006854) | AR | N/A | osteogenesis imperfecta type 21 |
| PMS2  (NM_000535) | AD, AR | N/A | Lynch syndrome 4, mismatch repair cancer syndrome 4 |
| AP5Z1  (NR_040253) | AR | N/A | hereditary spastic paraplegia 48 |
| PRKAR1B  (NM_001164760) | AD | N/A | MarbachSchaaf neurodevelopmental syndrome |
| DNAAF5  (NM_017802) | AR | N/A | primary ciliary dyskinesia 18 |
| WIPI2  (NM_015610) | AR | N/A | intellectual developmental disorder with short stature and variable skeletal anomalies |
| EIF2AK1  (NM_014413) | AD | N/A | leukoencephalopathy motor delay spasticity and dysarthria syndrome |
| INTS1  (NM_001080453) | AR | N/A | neurodevelopmental disorder with cataracts poor growth and dysmorphic facies |
| MAD1L1  (NM_001013836) | AR | N/A | familial prostate carcinoma, mosaic variegated aneuploidy syndrome 7 with inflammation and tumor predisposition |
| LFNG  NM_001040167 | AR | N/A | spondylocostal dysostosis 3 autosomal recessive |
| FAM20C  (NM_020223) | AR | N/A | lethal osteosclerotic bone dysplasia |
| AIMP2  (NM_006303) | AR | N/A | leukodystrophy hypomyelinating 17 |
| LOC100131257 (NR_034022) | N/A | N/A | - |
| CYTH3  (NM_004227) | N/A | N/A | - |
| SNORA80D (NR_145771) | N/A | N/A | - |
| LOC116435278  (NR_165238) | N/A | N/A | - |
| ZFAND2A-DT  (NR_110068) | N/A | N/A | - |
| CCZ1  (NM_015622) | N/A | N/A | - |
| MICALL2  (NR_106726) | N/A | N/A | - |
| C7orf26 (NM_024067) | N/A | N/A | - |
| MIOS  (NM_019005) | N/A | N/A | - |
| RNF216P1  (NR_015449) | N/A | N/A | - |
| LOC100128653  (NR_149036) | N/A | N/A | - |
| TNRC18  (NM_001080495) | N/A | N/A | - |
| RPA3  (NM_002947) | N/A | N/A | - |
| GRID2IP  (NM_001145118) | N/A | N/A | - |
| RSPH19B2  (NM_001099697) | N/A | N/A | - |
| ZNF8909P  (NR_034163) | N/A | N/A | - |
| DAGLB  (NM_139179) | N/A | N/A | - |
| TMEM184A  (NM_001097620) | N/A | N/A | - |
| RADIL  (NM_018059) | N/A | N/A | - |
| ZNF316  (NM_001278559) | N/A | N/A | - |
| SNX8  (NM_013321) | N/A | N/A | - |
| GNA12  (NM_007353) | N/A | N/A | - |
| GDI2  (NM_001494) | N/A | N/A | - |
| BRAK-RBAKDN  (NM_001204513) | N/A | N/A | - |
| MIR6874  (NR_106934) | N/A | N/A | - |
| LOC105375131  (NR_158215) | N/A | N/A | - |
| PDGFA  (NM_033023) | N/A | N/A | - |
| GPR146  (NM_001303473) | N/A | N/A | - |
| CYP2W1  (NM_017781) | N/A | N/A | - |
| RSPH10B  (NM_173565) | N/A | N/A | - |
| LOC101927354  (NR_108073) | N/A | N/A | - |
| EIF3B  (NM_001037283) | N/A | N/A | - |
| FAM220A  (NM_001037163) | N/A | N/A | - |
| MIR4655  (NR_039799) | N/A | N/A | - |
| C1GALT1  (NM_020156) | N/A | N/A | - |
| ZNF12  (NM_016265) | N/A | N/A |  |
| PRKAR1B-AS2  (NR_132384) | N/A | N/A |  |
| MIR4648  (NR_039791) | N/A | N/A | - |
| PSMG3-AS1  (NR_027329) | N/A | N/A | - |
| LOC112267991  (NR_165236) | N/A | N/A | - |
| ELFN1-AS1  (NR_120509) | N/A | N/A |  |
| MIR589  (NR_030318) | N/A | N/A |  |
| FSCN1  (NM_003088) | N/A | N/A | - |
| GPER1  (NM_001098201) | N/A | N/A | - |
| COX19  (NM_001031617) | N/A | N/A | - |
| PMS2CL  (NR_002217) | N/A | N/A |  |
| ADAP1  (NM_006869) | N/A | N/A |  |
| USP42  (NM_032172) | N/A | N/A | - |
| LOC442497  (NR_033960) | N/A | N/A | - |
| ANKRD61  (NM_001271700) | N/A | N/A | - |
| ZDHHC4  (NM_001134389) | N/A | N/A |  |
| UNCX  (NM_001080461) | N/A | N/A |  |
| FOXL3-OT1  (NR_164665) | N/A | N/A | - |
| COL28A1  (NM_001037763) | N/A | N/A | - |
| RNF216-IT1  (NR_046834) | N/A | N/A | - |
| MMD2  (NM_198403) | N/A | N/A |  |
| MIR6836  (NR_132384) | N/A | N/A |  |
| FAM220A  (NM_001037163) | N/A | N/A | - |
| MIR4655  (NR_106895) | N/A | N/A | - |
| PAPOLB  (NM_020144) | N/A | N/A | - |
| ZNF12  (NM_016265) | N/A | N/A |  |
| SDK1  (NM_152744) | N/A | N/A |  |
| C7orf50  (NM_001318252) | N/A | N/A | - |
| FOXL3  (NM_001374838) | N/A | N/A | - |
| PSMG3  (NM_032302) | N/A | N/A | - |
| LOC105375115  (NR_134324) | N/A | N/A |  |
| HRAT92  (NR_033963) | N/A | N/A |  |
| LOC100129603  (NR_126024) | N/A | N/A |  |
| PRKAR1B-AS1  (NR_110055) | N/A | N/A | - |
| LOC221946  (NR_126168) | N/A | N/A | - |
| RBAKDN  (NR_015343) | N/A | N/A | - |
| GRIFIN  (NM_001291784) | N/A | N/A |  |
| LOC105375303  (NR_165252) | N/A | N/A |  |
| TFAMP1  (NR_001288) | N/A | N/A | - |
| MAFK  (NM_002360) | N/A | N/A | - |
| MIR339  (NR_029898) | N/A | N/A | - |
| SNORD165  (NR_145804) | N/A | N/A |  |
| AMZ1  (NM_001384743) | N/A | N/A |  |
| SNORA114  (NR_145797) | N/A | N/A | - |
| MIOS-DT  (NR_110084) | N/A | N/A | - |
| ELFN1  (NM_001128636) | N/A | N/A | - |
| OCM  (NM_001097622) | N/A | N/A |  |
| SUN1  (NM_001130965) | N/A | N/A |  |
| TTYH3  (NM_025250) | N/A | N/A |  |
| CCZ1B  (NM_198097) | N/A | N/A | - |
| CHST12  (NM_018641) | N/A | N/A | - |
| SLC29A4  (NM_153247) | N/A | N/A | - |
| NUDT1  (NM_002452) | N/A | N/A |  |
| ZFAND2A  (NM_182491) | N/A | N/A |  |
| FOXK1  (NM_001037165) | N/A | N/A | - |
| ZNF815P  (NR_023382) | N/A | N/A | - |
| FBXL18  (NM_024963) | N/A | N/A | - |
| RBAK  (NM_021163) | N/A | N/A |  |
| MIR3683  (NR_037454) | N/A | N/A |  |
| ZNF853  (NM_017560) | N/A | N/A | - |
| LOC100129484  (NM_001354886) | N/A | N/A | - |
| UMAD1  (NM_001302348) | N/A | N/A | - |

N/A not available; AR autosomal recessive; AD autosomal dominant
